# Typical Crop Classification of Agricultural Multispectral Remote Sensing Images by Fusing Multi-Attention Mechanism ResNet Networks

**DOI:** 10.3390/s25072237

**Published:** 2025-04-02

**Authors:** Zongpu Li, Zhiyun Xiao, Yulong Zhou, Tengfei Bao

**Affiliations:** 1 Inner Mongolia Key Laboratory of Electrical and Mechanical Control, Inner Mongolia University of Technology, Hohhot 010080, China; 20221800545@imut.edu.cn (Z.L.); 20221100441@imut.edu.cn (Y.Z.); 2 Intelligent Energy Technology and Equipment Engineering Research Center of Colleges and Universities of Inner Mongolia Autonomous Region, Inner Mongolia University of Technology, Hohhot 010080, China; 3 School of Electric Power, Inner Mongolia University of Technology, Hohhot 010080, China; 4 School of Electronic Information and Electrical Engineering, Shanghai Jiao Tong University, Shanghai 200240, China; btflmqy1314@sjtu.edu.cn

**Keywords:** deep learning, multispectral images, crop classification, residual ResNet network, attention mechanism

## Abstract

Traditional crop classification methods have three critical limitations: (1) dependency on labor-intensive field surveys with limited spatial coverage, (2) susceptibility to human subjectivity during manual data collection, and (3) the inability to capture fine-grained spectral variations due to the lack of multispectral analysis. This research introduces an enhanced crop classification and identification model based on a residual ResNet network. This model leverages multispectral remote sensing images from unmanned aerial vehicles (UAVs) to accurately classify complex crop planting structures. The research focuses on four typical crops: sunflower, corn, beet, and pepper. By acquiring and preprocessing multispectral remote sensing image data, an improved ResNet50 model integrating the ACmix self-attention module and a coordinate attention mechanism is developed to enhance the classification and recognition accuracy of these crops. Experimental results demonstrate that the improved model achieves a classification accuracy of 97.8% on multispectral images, outperforming both RGB images and traditional methods. This research highlights the potential of combining UAV multispectral remote sensing technology with deep learning for precise crop classification, offering valuable technical support for precision agriculture management.

## 1. Introduction

Crop classification and identification involve in-depth analytical studies of crop images to provide theoretical and data foundations for precise scientific crop management. Traditional methods rely on field surveys to gather information on crop types and planting areas, using statistical data and sampling techniques for verification. These methods aim to determine changes in crop distribution and characteristics based on survey results. However, they are statistically dependent, prone to investigator subjectivity, labor-intensive, and time-consuming. Moreover, these approaches introduce errors and lack spatially distributed representations of crop classification results. For instance, field surveys often miss intra-field variability caused by microclimatic conditions, while satellite-based approaches lack the required temporal resolution for dynamic crop monitoring. These gaps necessitate a hybrid approach combining UAV multispectral imaging and deep learning.

Remote sensing technology can identify the spectral information of different features because the internal structures of leaves vary among crops, influencing their reflective spectral characteristics. By analyzing remote sensing images, distinct spectral and texture features of different crops can be identified. These differences in spectral characteristics enable the classification of crop types and the determination of their spatial distribution and other planting information. Remote sensing image analysis is therefore crucial for acquiring accurate crop classification and identification data.

Recent advancements in UAV multispectral technology have been widely adopted in precision agriculture globally. In China, researchers have developed crop monitoring systems using DJI drones equipped with RedEdge-MX sensors, achieving over accuracies of 85% in rice and wheat classification. Internationally, studies leveraging Sentera 6X sensors on fixed-wing UAVs demonstrate similar success in soybean and corn differentiation. However, challenges persist in handling spectral redundancy and spatial heterogeneity, particularly for crops with overlapping phenological stages.

The quality of crop image data sources and the choice of classification methods directly impact the accuracy of crop classification, identification, and subsequent yield estimation. In recent years, unmanned aerial vehicle (UAV) technology has demonstrated broad application prospects in civil domains. Currently, UAVs are utilized in over 40 civil fields, including agricultural plant protection, express delivery, map mapping, and film production. Since the early 21st century, agricultural UAVs have been widely adopted for crop classification and extraction. For instance, Hassan et al. evaluated the land use/land cover (LULC) characteristics of Penang Island using images captured by a fixed-wing UAV, achieving promising results [1]. While satellite remote sensing remains the primary method for large-scale crop monitoring due to its high costs and data access cycles, UAV remote sensing offers a cost-effective, efficient, and user-friendly alternative, making it particularly valuable for small- to medium-scale crop monitoring applications.

Traditional crop classification methods, such as manual field surveys and satellite-based monitoring, face critical limitations in practical agricultural applications. Field surveys are inherently labor-intensive and suffer from spatial sampling bias, while satellite imagery struggles with insufficient temporal resolutions and cloud interference. These methods fail to capture the dynamic spectral variations of crops during growth stages, leading to delayed decision-making in precision agriculture.

Crop classification methods have diversified significantly in recent years, driven by advancements in deep learning technology and improvements in algorithmic performance. These developments have led to substantial progress in intelligent agriculture. For example, Dai et al. classified four farmland features—cotton, corn, alfalfa, and zucchini—using a visible remote sensing classifier on a drone, achieving an overall accuracy of nearly 80.00% [2]. Zhao et al. employed UAV multispectral remote sensing to classify three types of weeds, achieving a correct identification rate of 96.3% [3]. These studies highlight the effectiveness of feature extraction methods that integrate spectral, texture, and spatial features, combined with neural networks and deep learning technologies. Such approaches enable end-to-end automation with minimal manual intervention, facilitating efficient crop information extraction. Current deep learning models, such as VGG and Efficient Net, often underperform in multispectral crop classification due to their inability to jointly model spectral–spatial dependencies. Additionally, conventional attention mechanisms lack positional awareness, limiting their effectiveness in distinguishing crops with similar spectral signatures. The integration of UAV multispectral imaging with deep learning addresses these gaps by enabling high-frequency, high-resolution data acquisition and automated feature extraction. This approach is particularly crucial for regions with fragmented farmlands and diverse crop rotations, where rapid and accurate classification is essential for yield prediction and resource allocation.

We trained a deep learning model based on the ResNet architecture on multispectral images for crop classification and recognition. Using Python 3.8.16 and the Pytorch 1.13.1 deep learning framework, the model is designed to minimize parameters and computational requirements. Ground truth crop labels are combined with feature map analysis to construct a training sample library for four crops: sunflower, maize, sugar beet, and chili pepper. The model processes training and validation set images, enhancing feature extraction through an attention mechanism that focuses on distinguishing the features of specific crops. By assigning different weights to features or feature channels, the attention mechanism emphasizes critical information while suppressing less relevant data. This strategy not only improves model performance but also enhances interpretability and generalization capabilities, resulting in an optimized ResNet model tailored for crop classification and recognition.

This study addresses the crop classification problem using UAV multispectral images as data sources. Spectral features are extracted and optimal feature parameters for each crop are identified. Different classification models are employed to determine the distribution of crop planting information. The research begins with UAV remote sensing image acquisition in the research area, followed by image stitching and preprocessing. A deep learning neural network model is then applied to classify and identify crops, enabling the timely assessment of crop areas and their distribution. The primary contributions of this study are threefold: (1) a novel ResNet50 architecture integrating ACmix self-attention and coordinate attention mechanisms, specifically designed to capture both global spectral context and local spatial features; (2) a systematic framework for UAV multispectral data acquisition and fusion, optimized for complex planting structures in arid regions; and (3) comprehensive validation demonstrating superior performance (97.8% accuracy) compared to state-of-the-art models including the Vision Transformer and Efficient Net variants. This study aims to explore the potential of UAV multispectral remote sensing for crop extraction in regions with complex planting structures and to compare the effectiveness of various classification methods. The findings are expected to provide technical support for agricultural production and expand the application scope of UAV technology. Our proposed model addresses these challenges by integrating an ACmix self-attention module and coordinate attention mechanism into ResNet50, enabling the simultaneous extraction of local spectral features and global contextual dependencies.

## 2. Materials and Methods

### 2.1. Data Acquisition and Overview of the Research Area

**Figure 1:**
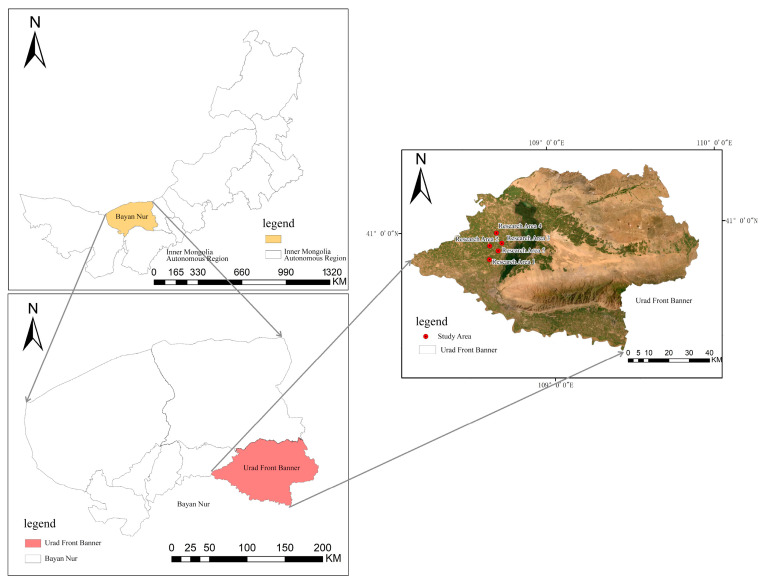
Geographic location of the research area (40°56′ N, 108°52′ E).

In this study, the crop planting areas within the Inner Mongolia Autonomous Region were selected as the research focus, encompassing five distinct study sites. All of these sites are situated in Ulatqian Banner, Bayannur City, within the Inner Mongolia Autonomous Region, as illustrated in Figure 1. The region features a complex and diverse crop planting structure, offering an ideal setting to investigate the application of UAV remote sensing in planting structure extraction.

The research areas are situated around the Wuliangsu Sea (40°56′ N, 108°52′ E). Research area 1 comprises sunflowers and chili peppers, area 2 maize and sugar beet, area 3 chili peppers with a disturbance category, and areas 4 and 5 consist of maize and sunflowers. The region exhibits a typical mesothermal continental monsoon climate, with abundant sunshine, dry conditions, and frequent winds. The multi-year average total solar radiation is 6424.2 MJ/m². Annual precipitation ranges from 130 to 285 mm, primarily concentrated during summer and fall, creating favorable conditions for crop growth. The area benefits from ample water resources, sufficient for conventional crops. The frost-free period lasts from 117 to 136 days and is relatively short, which helps prevent issues such as excessive crop growth, delayed maturity, and reduced yields. This climatic pattern promotes concentrated crop growth and contributes to abundant harvests.

### 2.2. Unmanned Aerial Vehicle Data

The experimental data used in this study were obtained from a DJI industry-grade UAV, the M300RTK (Shenzhen DJI Innovation Technology Co., Shenzhen, China), equipped with a multispectral gimbal, the MS600Pro (Changguang Yuchen Information Technology and Equipment (Qingdao) Co., Qingdao, China). The MS600Pro has a spatial resolution of 1.3 M pixels and captures a spectral range of 400–1000 nm, with band ranges of 450 nm, 555 nm, 660 nm, 720 nm, 750 nm, and 840 nm.

The UAV is equipped with a satellite navigation system that can be used to autonomously plan flight paths within the study area. Table 1 lists the details of the UAV equipment.

Data acquisition was conducted between 15–20 August 2023, during the peak growth stage of crops. Flights were scheduled between 10:00 a.m. and 2:00 p.m. under clear sky conditions (cloud cover <10%) to minimize solar angle variations and ensure stable drone photography. The ground resolution of aerial photography was set to 6 cm, with an altitude of 80 m for aerial flight and 120 m for return flight. The vehicle’s flight speed for UAV image acquisition was set to 15 m/s, and the spatial resolution of UAV data was 2.5 cm. The flight image overlap rate was set to 70% in the heading direction and 65% in the inter-flight direction. Photographs were taken along the S-route in the study area using flight paths.

### 2.3. Unmanned Aerial Vehicle Multispectral Image Data Acquisition and Preprocessing

In this study, we leverage the advantages of UAV remote sensing technology for the extraction of crop cultivation information to classify crops in agricultural areas with highly complex planting structures in Ulatqian Banner, Bayannur City, Inner Mongolia. We generate crop distribution maps for each research site and conduct comparative analyses to evaluate the feasibility and applicability of different classification models.

Multispectral data sampling in the research area was conducted using the DJI industry-grade UAV M300RTK, equipped with the MS600Pro multispectral gimbal to collect sample data images. Since the spectral characteristics of crops vary significantly under the same phenological period but different lighting conditions, we took measures to minimize external influences. To avoid the impact of changing solar angles and shading from clouds or other objects on light intensity, and to ensure consistency in phenological stages and lighting conditions during image acquisition, we selected a clear day with ground wind speeds below level 4 for the drone data acquisition experiment. During this time, the sun’s radiation angle was low, resulting in minimal ground shadows, which ensured high-quality image data.

**Figure 2:**
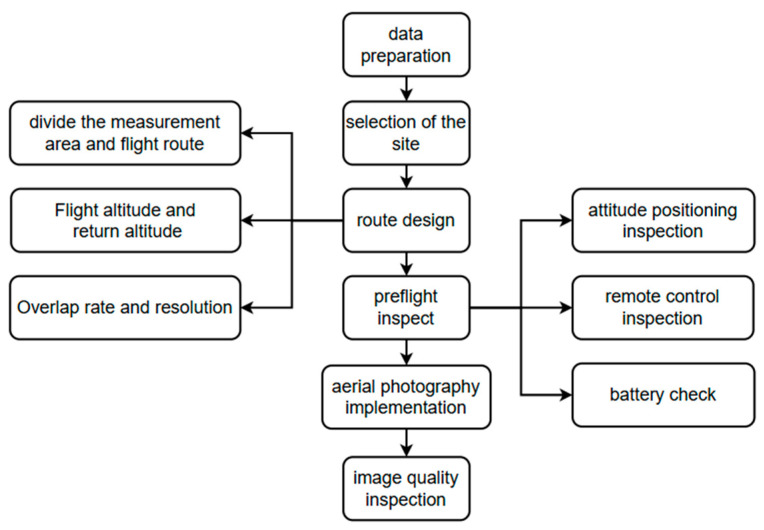
Technical routes for the acquisition of imagery by unmanned aerial remote sensing.

The technical workflow for UAV remote sensing image acquisition in this study is illustrated in Figure 2.

Before image capture, whiteboard correction is performed to minimize distortions caused by uneven light intensity. Prior to take-off, the rotor blades are properly installed, and the frequency alignment of the UAV is conducted to ensure the normal operation of its navigation system. Additionally, the existing satellite image map of the research area is imported into the UAV flight control system. Based on the remote sensing image map, the area is divided into regions, and the flight route is designed with pre-calculated flight parameters. The flight altitude is set to 80 m, with a heading overlap of 70% and a lateral overlap of 65%, to reduce errors caused by lighting variations. Field research data and actual measurement results are used as auxiliary data, and the collected data undergo preprocessing.

Post-flight, aerial photography data, susceptible to weather conditions and other factors causing unstable camera positioning, were subject to quality checks. Original UAV aerial images exported from the onboard memory card were inspected, and based on the results of the quality check, a decision was made on whether to re-fly the mission or implement a supplementary flight plan, depending on the proportion of non-compliant images relative to the total aerial footage.

**Figure 3:**
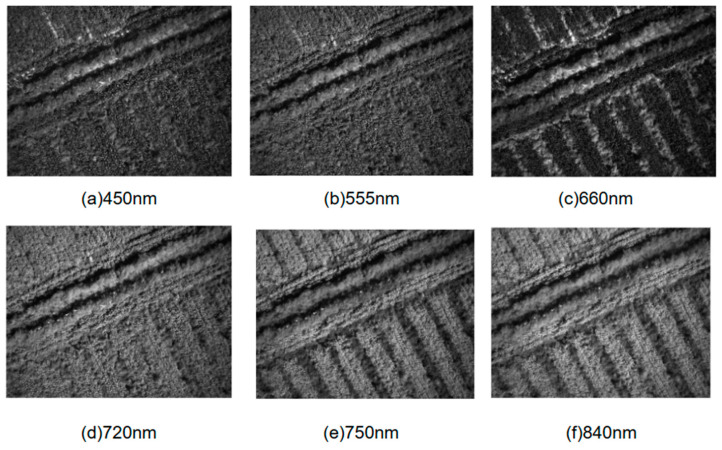
UAV six-channel multispectral image of a photo site in the research area (all images are displayed at native resolution).

Finally, a six-channel multispectral image of the UAV at a specific photo point in the research area was obtained, as illustrated in Figure 3.

Unmanned aerial vehicle (UAV) remote sensing technology utilizes UAVs as airborne platforms, equipped with various micro-sensors, to collect electromagnetic wave information on ground features at low altitudes. This approach leverages the unique advantages of UAVs, enabling the acquisition of high-resolution remote sensing images. By analyzing these images, it becomes possible to reveal the changing patterns of features and their inter-relationships.

**Figure 4:**
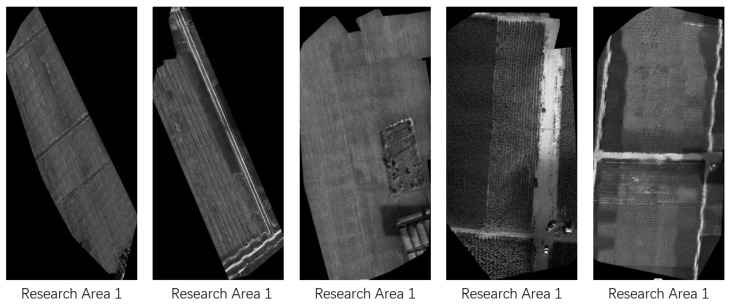
UAV orthophoto of the research area (all images are displayed at native resolution).

In this study, we utilize a rapid production method for UAV orthophotos without requiring control point data. The data processing system Pix4D Mapper (version 4.5.6) is used for integrated orthophoto generation. Pix4D does not necessitate the addition of ground control points during processing, as it can estimate geographic positions based on the UAV’s built-in GPS. The resulting UAV orthophoto of the research area, clipped according to the defined boundaries, is shown in Figure 4.

This study achieves image fusion through image stitching and channel fusion. Stitching fusion in the image channel dimension combines different color channels from multiple images to create a new composite channel image, which serves as an enhanced input for processing. This approach allows the network to simultaneously consider a broader range of visual information, improving the model’s ability to interpret and represent image content. To enhance image quality and information richness, six-channel images from UAV orthophoto maps of the research area were fused. This fusion process not only improves recognition accuracy but also compensates for the limitations of individual crop features, providing robust support for enhancing the classification and recognition model.

**Figure 5:**
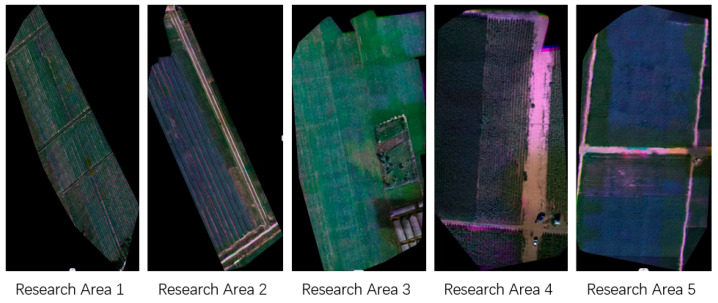
Six-channel pseudo-color image of a UAV orthophoto of the research area (all images are displayed at native resolution).

The six-channel pseudo-color map of the UAV orthophoto image in the research area is illustrated in Figure 5.

## 3. Methodology

In this study, we utilize an enhanced ResNet network to extract crops from agricultural areas with varying levels of planting structure complexity, leveraging the spatial structure of the data. This approach aims to explore the potential of UAV multispectral remote sensing technology combined with deep learning algorithms for accurately identifying crops in regions with diverse planting patterns.

**Figure 6:**
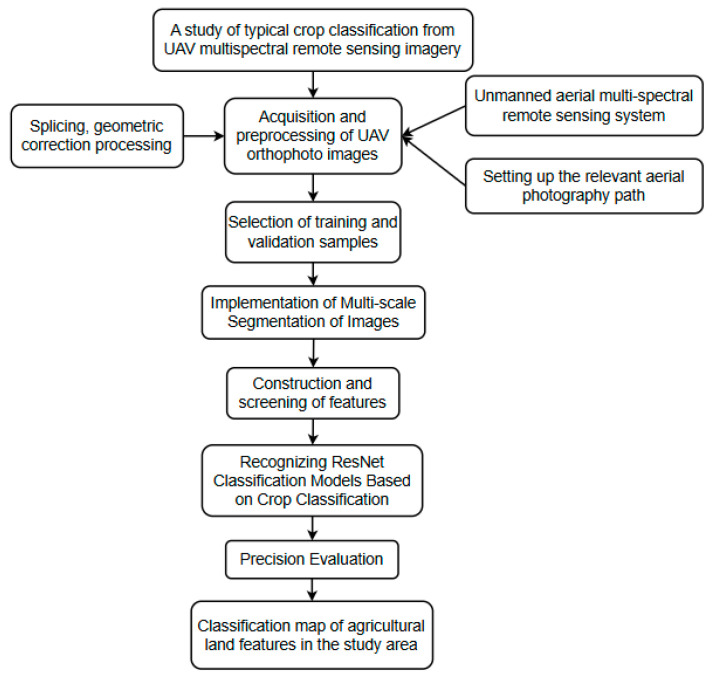
Flow chart of agricultural land feature extraction based on multispectral remote sensing.

The workflow for typical crop extraction in this study, as illustrated in Figure 6, includes the following steps:

(1) Acquisition and preprocessing of UAV remote sensing images: this involves setting up the UAV multispectral system, designing aerial photography paths, and performing image stitching and geometric correction to generate orthophoto images.
(2) Generation of training and validation samples: UAV multispectral images are processed to create labeled datasets for model training and validation.
(3) Multi-scale image segmentation: images are segmented at multiple scales to capture detailed spatial and spectral features.
(4) Feature construction and screening involve identifying and optimizing distinctive features of different crops to enhance classification accuracy.
(5) Image classification using the improved ResNet model: the enhanced ResNet model is applied to classify farm crops based on the extracted features.
(6) Accuracy evaluation: the performance of the model is assessed using a confusion matrix, enabling a comparative analysis of classification accuracy across different models.

This comprehensive approach ensures robust crop extraction and classification, demonstrating the effectiveness of integrating UAV multispectral remote sensing with advanced deep learning techniques.

### 3.1. Improved ResNet50 Classification Model Based on Crop Classification and Recognition

Convolutional neural networks (CNNs) are a cornerstone algorithm in deep learning and artificial intelligence, extensively applied in fields such as image recognition and natural language processing. However, traditional CNNs face challenges as network depth increases, often encountering issues such as gradient vanishing or gradient explosion, which degrade model performance.

ResNet50, a deep convolutional neural network-based image classification algorithm, addresses these limitations [4,5,6]. Proposed by Kaiming He et al. from Microsoft Research, ResNet50 is a prominent member of the ResNet family. Compared to traditional CNN models, ResNet50 features a deeper network structure and introduces residual connections to mitigate gradient vanishing during training, significantly enhancing model performance. Its balanced depth and excellent convergence properties have made it widely adopted in various applications.

A comparison of the parameters of the ResNet series is provided in Table 2.

**Figure 7:**
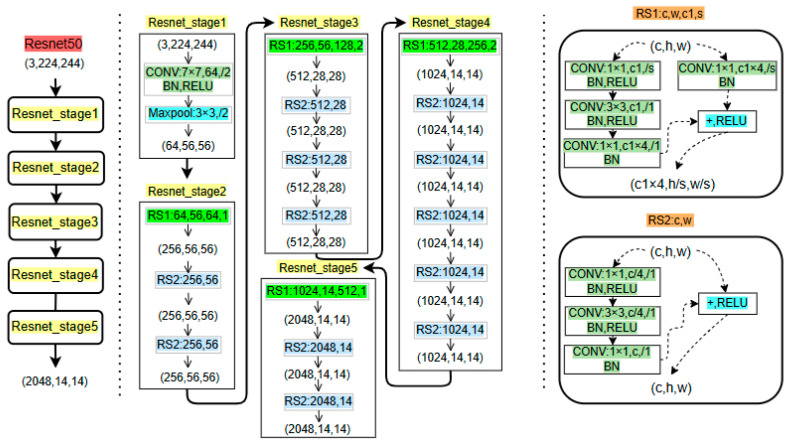
Schematic diagram of the ResNet50 network structure. Parameters: c (channels), h (height), w (width), and s (stride).

The ResNet50 architecture, employed in this study, comprises five distinct stages for feature extraction: an initial convolutional processing block followed by four residual blocks. The convolutional processing block performs preliminary feature extraction and input dimension transformation through four sequential components: a 7 × 7 convolutional layer, a batch normalization (BN) layer, a ReLU activation layer, and a 3 × 3 max pooling layer. The subsequent four residual blocks contain varying numbers of residual units: three, four, six, and three, respectively. These residual units are categorized into two types—the dimension-altering residual structure (RS1) and the dimension-preserving residual structure (RS2) [7,8]. This strategic implementation of these two residual structures enables ResNet50 to effectively address the vanishing gradient problem while achieving superior feature extraction performance. The detailed architectural configuration of the ResNet50 network is illustrated in Figure 7.

### 3.2. ACmix Self-Attention Module

Multispectral data’s high-dimensional characteristics offer rich feature information across various applications, with each spectral band representing distinct physical and chemical properties. However, this increased dimensionality simultaneously introduces the challenges of feature redundancy and noise interference, complicating data processing for analytical models. Traditional CNNs often exhibit limitations in capturing global features and long-range dependencies when processing multispectral data, primarily due to their complex dimensionality and extensive feature information. The integration of the ACmix self-attention module into the ResNet50 architecture significantly enhances the model’s feature representation capability, robustness, and adaptability [9], effectively addressing the limitations of conventional residual networks in feature extraction [10]. This innovative combination presents a novel approach for handling complex multispectral data.

The ACmix attention mechanism represents a groundbreaking fusion methodology that synergistically combines the strengths of self-attention and convolution operations [11], which are specifically designed to enhance computer vision model performance. This module substantially improves multispectral data analysis through its dynamic learning capability, which effectively assesses feature importance while simultaneously capturing long-range dependencies across different spectral bands.

**Figure 8:**
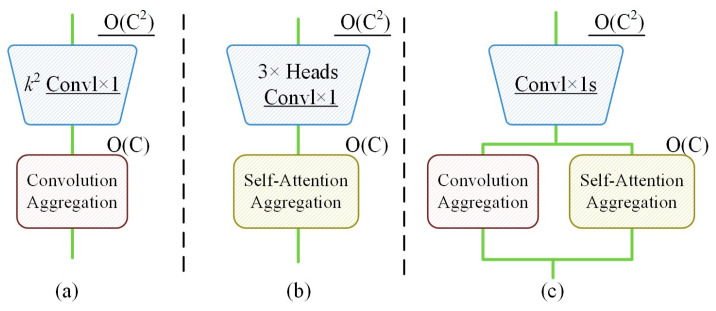
Comparison of computational complexity of feature channels: (**a**) computational complexity of conventional convolution; (**b**) computational complexity of self-attention; (**c**) computational complexity of the ACmix attention mechanism.

Convolution and self-attention, traditionally seen as distinct paradigms in representation learning, exhibit a profound underlying connection through ACmix’s innovative approach. The module reveals that conventional convolution operations can be decomposed into a series of 1 × 1 convolutions combined with shifting and summation operations for feature extraction. Similarly, the projection processes of queries, keys, and values in self-attention mechanisms can be interpreted as multiple 1 × 1 convolution operations. This conceptual unification provides valuable insights into the fundamental relationships between these two computational paradigms. The comparative analysis of computational complexity across feature channels is illustrated in Figure 8.

As a prominent attention mechanism, self-attention fundamentally comprises three essential components: query, key, and value [12]. In computer vision applications, the self-attention module enhances feature representation by computing and aggregating global contextual information across the entire image [13], thereby achieving a larger receptive field compared to conventional convolution operations. This architectural similarity in the initial processing stage between self-attention and convolution operations establishes a theoretical foundation for their effective integration.

The core innovation of ACmix lies in its sophisticated fusion of self-attention and convolution paradigms through a hybrid architecture while maintaining computational efficiency. The ACmix module employs eight attention heads with a spatial reduction ratio of 4, balancing computational efficiency and feature discriminability. The hybrid path weights (α = 0.6 for convolution, β = 0.4 for attention) were optimized through grid search on the validation set. The mechanism initially processes input feature maps through 1 × 1 convolutions to generate intermediate features, which are subsequently processed through parallel pathways corresponding to both self-attention and convolution operations. This dual-path architecture enables ACmix to simultaneously leverage the global contextual understanding of self-attention mechanisms and the local feature extraction capabilities of convolutional operations, thereby enhancing model performance without significantly increasing computational complexity.

The ACmix attention mechanism offers substantial advantages in computer vision applications through its synergistic combination of self-attention and convolution benefits. This integration significantly improves model performance in feature extraction while maintaining computational efficiency [14,15,16], making it particularly suitable for practical implementations. The mechanism demonstrates remarkable versatility across various computer vision tasks, including image classification, object detection, and semantic segmentation. Its implementation combines simplicity with effectiveness, consistently delivering superior performance across diverse applications. As deep learning technology continues to evolve, ACmix is poised for broader adoption and further development in the field of computer vision.

**Figure 9:**
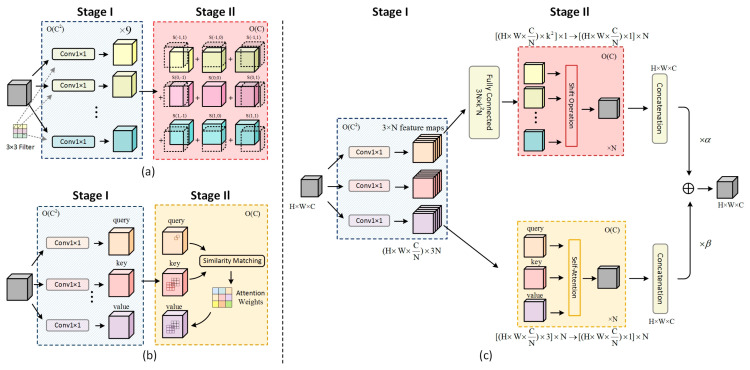
Schematic diagram of the ACmix self-attention module’s network structure: (**a**) conventional convolution: kernel size k×k, input channels c, height h, width w, and stride s; (**b**) self-attention: query (Q), key (K), and value (V) matrices for global context modeling; (**c**) ACmix hybrid architecture: combines 1 × 1 convolutions (blue arrows) and attention weight computation (orange arrows), where ⊗ denotes element-wise multiplication.

The architectural schematic of the ACmix self-attention module is presented in Figure 9, illustrating its innovative network structure.

### 3.3. Coordinate Attention

**Figure 10:**
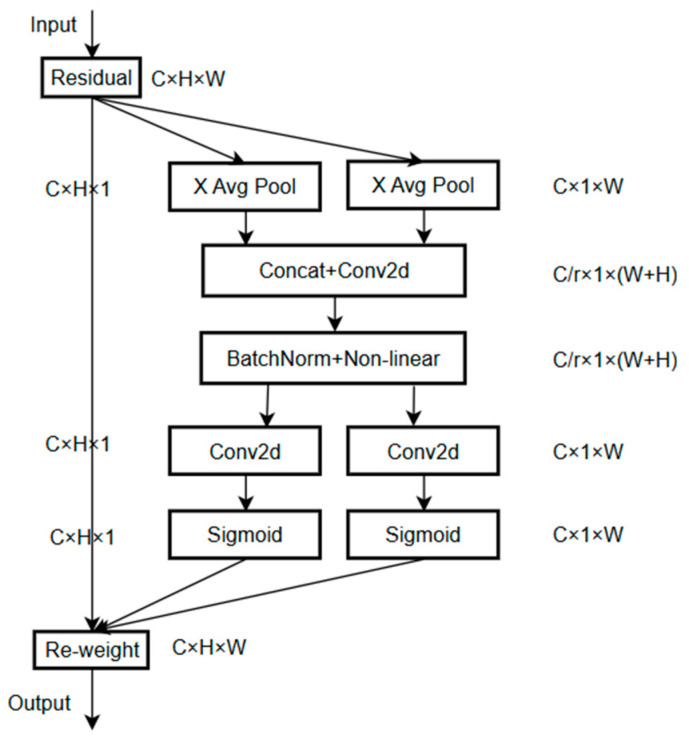
Schematic diagram of the coordinate attention mechanism. X: input feature map; H×W: spatial dimensions; C: channels; ⊗: convolution operation.

Coordinate attention is a novel mechanism that strategically incorporates precise spatial positional information into neural network architectures [17,18], enhancing the model’s ability to capture both channel relationships and long-range dependencies. This mechanism is particularly effective in lightweight network architectures, offering significant performance improvements while maintaining computational efficiency. Coordinate attention is implemented through two distinct yet complementary stages, coordinate information embedding and coordinate attention generation, which work together to optimize feature representation. The architectural details and operational flow of the coordinate attention mechanism are illustrated in Figure 10.

In the coordinate information embedding phase, spatial location information is systematically integrated into the input feature map. Departing from conventional global pooling approaches, coordinate attention innovatively decomposes the global pooling operation into two distinct one-dimensional feature encoding processes. These processes independently encode each channel along the horizontal and vertical axes, enabling the capture of long-range dependencies in one spatial dimension while preserving precise positional information in the orthogonal dimension. Specifically, for input feature map X, specialized pooling kernels with dimensions (H,1) and (1, W) are employed to perform features encoding along the horizontal and vertical directions, thereby generating two direction-sensitive feature representations.

Coordinate attention incorporates a channel reduction factor of 16, compressing the input feature map from C to C/16 channels before spatial encoding. This configuration reduced FLOPs by 38% compared to standard SE blocks while maintaining >95% accuracy. The coordinate attention generation phase utilizes these direction-aware feature maps to construct a comprehensive attention mechanism. The process begins with the concatenation of the two directional feature maps, followed by a convolutional transformation to produce an intermediate feature representation. This intermediate feature map is then partitioned along the spatial dimension into two separate tensors, each undergoing independent 1 × 1 convolutional transformations to derive attention weights for their respective directions. These computed attention weights are applied to the original input feature map, effectively enhancing the representation of spatially significant features. This sophisticated attention mechanism incorporates not only inter-channel relationships but also direction-sensitive and location-aware information, significantly improving the model’s capability for precise target localization and recognition.

Coordinate attention represents a significant advance in attention mechanisms, offering an efficient solution for modeling channel relationships and long-range dependencies through its innovative integration of positional information within channel attention. Its architectural flexibility and computational efficiency make it particularly well-suited for deployment in lightweight network architectures. The mechanism’s ability to deliver substantial performance enhancements with minimal computational overhead establishes it as an ideal solution for optimizing model performance across a range of computer vision tasks.

### 3.4. General Structure of the Improved ResNet Network

The enhanced ResNet50 network architecture, incorporating both the ACmix self-attention module and coordinate attention mechanism, represents a significant advance over the classical ResNet50 framework [19,20,21,22,23,24]. This improved architecture is specifically designed to optimize performance in image classification and feature extraction tasks. The incorporation of these sophisticated attention mechanisms enables the network to more effectively identify and emphasize critical features, substantially improving overall model performance. The synergistic combination of traditional convolutional operations with modern attention mechanisms in this enhanced architecture results in superior feature extraction capabilities and classification accuracy [25]. Table 3 lists the impact of attention mechanisms on classification metrics.

**Figure 11:**
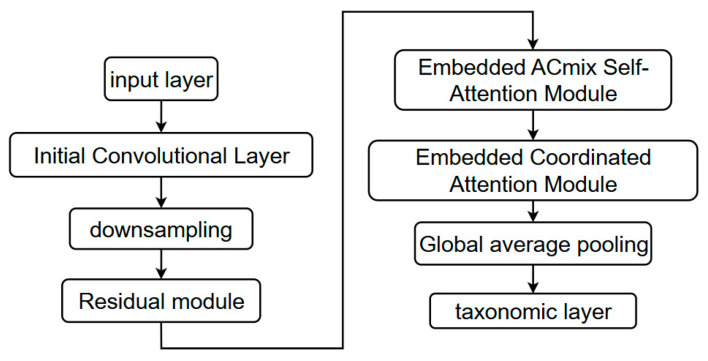
Flow chart of the overall structure of the improved ResNet network.

The architectural innovation lies in the strategic integration of ACmix’s hybrid self-attention–convolution approach with coordinate attention’s position-aware mechanism, creating a powerful feature extraction framework. This integration allows the network to simultaneously capture both local spatial features and global contextual relationships while maintaining precise positional information. The effectiveness of this enhanced architecture will be empirically validated through comprehensive experiments focused on crop classification and recognition tasks [26]. The complete architectural framework and data flow of the improved ResNet network are illustrated in Figure 11.

### 3.5. Evaluation Metrics

During the model evaluation phase, this study employs a comprehensive set of metrics derived from the confusion matrix to assess model performance. The evaluation framework incorporates precision (*P*), recall (*R*), *F*1 score, overall accuracy (*OA*), and the Kappa coefficient. These metrics provide a multi-faceted assessment of the model’s classification performance. The mathematical formulations for these evaluation metrics are presented as follows:

(1) $$P=\frac{TP}{TP+FP},$$

(2) $$R=\frac{TP}{TP+FN},$$

(3) $$F1=\frac{2*P*R}{P+R},$$

(4) $$OA=\frac{\sum TP+\sum TN}{\sum Total\;Samples},$$

(5) $$Kappa=\frac{OA-Pe}{1-Pe}$$

where *Pe* is the randomized consistency probability.

These metrics were selected to holistically assess model performance, balancing precision-recall trade-offs and overall consistency.

## 4. Results

### 4.1. Experimental Environment

In this study, the experimental code running environment is Python 3.8, the model training environment is based on PyTorch 1.13.1 [27], the experiments are carried out on a 64-bit Windows 10 system, the video memory is 16 GB, and the detailed configuration is shown in Table 4.

### 4.2. Dataset Production

The experimental data were acquired from a UAV multispectral remote sensing platform spanning five distinct research areas (research area 1 through research area 5) located in Ulatqian Banner, Bayannur City, Inner Mongolia Autonomous Region. This region exhibits a complex and diverse agricultural planting structure, providing an ideal environment for investigating crop pattern extraction using UAV remote sensing technology.

The data processing pipeline began with comprehensive preprocessing of the raw multispectral imagery, resulting in six-channel orthophoto maps for each research area. Subsequent processing involved spatial alignment and spectral channel fusion of the image data. The preprocessed imagery was then annotated using the LabelMe annotation tool, with each image meticulously labeled according to its respective crop category. The final curated image dataset was systematically partitioned into training and testing subsets in an 8:2 ratio, as detailed in Table 5.

### 4.3. Comparison Experiment

To validate the effectiveness of individual components in the proposed enhanced ResNet classification model for crop identification, a series of ablation experiments were conducted on the research area dataset. These experiments systematically evaluated the contributions of key modules, including improved multispectral remote sensing image processing, the ACmix self-attention module, and the coordinate attention mechanism. Ablation studies were conducted to quantify the contributions of each module. The baseline ResNet50 was incrementally augmented with ACmix and coordinate attention and tested under both multispectral (six-channel) and RGB (three-channel) inputs. Model performance was assessed through accuracy comparisons on the test set, with the presence of specific modules indicated by √ in Table 6, which presents the comprehensive results of the ablation study.

The experimental results demonstrate significant improvements in classification accuracy across different configurations. Comparative analysis between experiments 1–4 and 5–8 reveals that both the baseline ResNet50 and enhanced ResNet networks achieve substantially higher accuracy with multispectral six-channel images compared to RGB three-channel images, confirming the superiority of multispectral data for crop classification.

A detailed examination of the multispectral six-channel image experiments yields several key findings:

(1) A comparison between experiments 1 and 2 shows a 2.9% improvement in accuracy with the addition of the ACmix self-attention module.
(2) Experiments 1 and 3 demonstrate a 2.1% increase in accuracy following the integration of the coordinate attention mechanism.
(3) The combined implementation of both modules in experiment 4 yields a 4.7% accuracy enhancement, achieving the highest test set accuracy of 96.7%.

Quantitative analysis of Table 5 indicates that the proposed improved ResNet classification model consistently outperforms other configurations across all evaluation metrics, including precision (P), recall (R), F1 score, overall accuracy (OA), and the Kappa coefficient. The combined use of ACmix and coordinate attention achieved a 4.7% accuracy gain over the baseline, demonstrating their complementary roles in capturing spectral–spatial dependencies. These results substantiate the model’s superior performance in typical crop classification tasks.

To further validate the robustness and accuracy of the proposed model, comparative experiments were conducted against two established machine learning approaches: the object-based random forest (OB-RF) and object-based support vector machine (OB-SVM) classification models [28]. Random forest, known for its high prediction accuracy and robustness to outliers, requires optimization of two key parameters: the number of classification trees (ntree) and the number of features for node splitting (mtry). Support vector machines, based on statistical learning theory and structural risk minimization, excel in handling small sample sizes and high-dimensional pattern recognition problems [29], making them particularly suitable for remote sensing image classification.

Comparative experiments conducted on the same dataset reveal the classification accuracy of these models, as shown in Table 7. This provides comprehensive evidence of the proposed model’s superior performance.

To further validate the proposed model, we compared it against four benchmark architectures: VGG16, EfficientNet-B4, the Vision Transformer (ViT-B/16), and the Swin Transformer-Tiny. All models were trained on identical multispectral datasets with an input size of 224 × 224. Comparative experiments, conducted on the same dataset, reveal the classification accuracy of these models as shown in Table 8, providing comprehensive evidence of the proposed model’s superior performance.

The proposed model achieves 2.4% higher overall accuracy (OA) than the Swin Transformer while requiring 9% fewer parameters, demonstrating its efficiency in spectral–spatial feature fusion.

**Figure 12:**
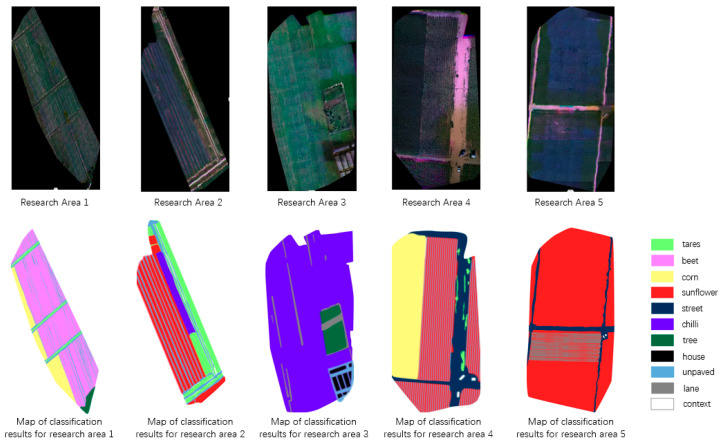
Categorical identification results map of the research area.

The classification results for the five research areas obtained by the enhanced Res-Net model proposed in this study are visually presented in Figure 12. The figure demonstrates the model’s performance in accurately classifying and recognizing different crop types across the various study regions.

## 5. Conclusions

This study proposes an enhanced ResNet classification model for crop identification built upon the ResNet50 architecture, integrating the ACmix self-attention module and incorporating the coordinate attention mechanism. The proposed model aims to investigate the potential and applicability of UAV multispectral remote sensing for complex agricultural pattern extraction, enabling more efficient and comprehensive feature extraction and significantly improving detection accuracy.

The research methodology involved acquiring UAV multispectral imagery from five research areas with varying planting structure complexities in Ulatqian Banner, Bayannur City, Inner Mongolia. These datasets enabled the exploration of UAV multispectral remote sensing technology’s potential for crop extraction in areas with complex planting structures. Comparative analyses were conducted against traditional machine learning approaches, including random forest and support vector machine classification models.

The experimental results demonstrate that the proposed improved ResNet classification model maintains consistent classification and extraction accuracy across research areas with different planting structure complexities. Notably, the model achieves an overall accuracy of 97.8% for classifying four distinct farmland features under highly complex planting structures, outperforming conventional multispectral remote sensing techniques in fine-grained agricultural classification tasks. While the model excels with six-channel multispectral data, its performance gain diminishes for RGB inputs (92.3% vs. 97.8%). This limitation stems from the ACmix module’s reliance on narrow-band spectral discriminability. Future work could explore adaptive channel pruning to maintain robustness across data modalities.

Building on current advancements in remote sensing image classification and deep learning research, this study presents a novel agricultural remote sensing framework: “UAV data acquisition plus deep learning-based big data analysis”. This innovative model combines UAV remote sensing technology with advanced deep learning techniques to achieve efficient and accurate crop classification. The proposed framework can be integrated with IoT-enabled edge devices for real-time crop monitoring. By coupling UAV data with soil moisture sensors and historical yield databases, farmers could implement dynamic irrigation strategies. Moreover, federated learning approaches could enable scalable deployment across distributed agricultural regions while preserving data privacy. The framework demonstrates superior classification accuracy and is particularly effective for the fine-grained classification of agricultural lands with complex cultivation patterns, representing a significant advancement in precision agriculture applications. 

## Figures and Tables

**Table 1:** DJI industry-grade UAV M300 RTK system parameters.

Parameters	Numerical Value
Volumetric	810 × 670 × 430 mm
Communications Radius	5000 m
Maximum Load	2700 g
Cruise Time	55 min
Maximum Take-off Weight	9000 g
Mapping Distance	15 km
Maximum Level Flight Speed	23 m/s
Propulsion	470 W
Maximum Wind Speed	15 m/s

**Table 2:** ResNet series’ related network parameters.

Layer Name	Output Size	18-Layer	34-Layer	50-Layer
Conv1	112 × 112	7 × 7, 64, stride2		
		3 × 3 max pool, stride2		
Conv2_x	56 × 56	$\left[\begin{array}{cc} 3\times 3, & 64\\ 3\times 3, & 64 \end{array}\right]$ × 2	$\left[\begin{array}{cc} 3\times 3, & 64\\ 3\times 3, & 64 \end{array}\right]$ × 3	$\left[\begin{array}{cc} \begin{array}{c} 1\times 1,\\ 3\times 3,\\ 1\times 1, \end{array} & \begin{array}{c} 64\\ 64\\ 256 \end{array} \end{array}\right]$ × 3
Conv3_x	28 × 28	$\left[\begin{array}{cc} 3\times 3, & 128\\ 3\times 3, & 128 \end{array}\right]$ × 2	$\left[\begin{array}{cc} 3\times 3, & 128\\ 3\times 3, & 128 \end{array}\right]$ × 4	$\left[\begin{array}{cc} \begin{array}{c} 1\times 1,\\ 3\times 3,\\ 1\times 1, \end{array} & \begin{array}{c} 128\\ 128\\ 512 \end{array} \end{array}\right]$ × 4
Conv4_x	14 × 14	$\left[\begin{array}{cc} 3\times 3, & 256\\ 3\times 3, & 256 \end{array}\right]$ × 2	$\left[\begin{array}{cc} 3\times 3, & 256\\ 3\times 3, & 256 \end{array}\right]$ × 6	$\left[\begin{array}{cc} \begin{array}{c} 1\times 1,\\ 3\times 3,\\ 1\times 1, \end{array} & \begin{array}{c} 256\\ 256\\ 1024 \end{array} \end{array}\right]$ × 6
Conv5_x	7 × 7	$\left[\begin{array}{cc} 3\times 3, & 512\\ 3\times 3, & 512 \end{array}\right]$ × 2	$\left[\begin{array}{cc} 3\times 3, & 512\\ 3\times 3, & 512 \end{array}\right]$ × 3	$\left[\begin{array}{cc} \begin{array}{c} 1\times 1,\\ 3\times 3,\\ 1\times 1, \end{array} & \begin{array}{c} 512\\ 512\\ 2048 \end{array} \end{array}\right]$ × 3
	1 × 1	Average pool, 1000-d fc, softmax		
FLOPs		1.8 × 10^9^	3.6 × 10^9^	3.8 × 10^9^

**Table 3:** Impact of attention mechanisms on classification metrics.

Module	Precision	Recall	F1 Score
Baseline ResNet	78.8	84.5	90.7
+ACmix	87.2 (+8.4)	88.4 (+3.9)	91.9 (+1.2)
+Coordinate Attention	85.3 (+6.5)	87.7 (+3.2)	91.2 (+0.5)

**Table 4:** Experimental environment parameters.

Project Environment	Configuration
Deep Learning Framework	Pytorch 1.13.1
Code Running Environment	Python 3.8.16
Display Card (computer)	NVIDIA GeForce RTX3090
CPU	AMD Ryzen Threadripper
CUDA Versions	12.4

**Table 5:** Image dataset composition.

Tabs	Crops	Training Sets (80%)	Test Sets (20%)	Aggregate
1	Beet	31,285	7821	39,106
2	Corn	5115	1279	6394
3	Sunflower	23,890	5973	29,863
4	Capsicum	22,122	5531	27,653
5	Other	75,232	18,808	94,040

**Table 6:** Ablation experiment.

Serial No.	Multispectral Six-Channel Images	RGB Three-Channel Image	Infrastructure Network ResNet50	ACmix Self-Attention Module	Coordinate Attention Mechanisms	Test Set Accuracy (%)	Precision (%)	Recall (%)	F1 Score (%)	Overall Accuracy (OA)	Kappa Factor (%)
1	√		√			83.5	78.8	84.5	90.7	93.6	90.3
2	√		√	√		91.5	87.2	88.4	91.9	97.3	94.9
3	√		√		√	89.0	85.3	87.7	91.2	96.9	96.0
4	√		√	√	√	97.8	92.1	94.8	93.5	98.8	97.5
5		√	√			82.1	74.7	81.2	90.1	92.1	86.9
6		√	√	√		90.7	85.4	86.0	91.8	96.5	90.8
7		√	√		√	85.7	83.9	84.7	90.6	96.6	90.1
8		√	√	√	√	92.3	90.5	91.3	92.4	97.2	95.9

**Table 7:** Comparison of experiments involving traditional algorithms.

Model	OA (%)	F1 Score	Params (M)	FLOPs (G)
VGG16	89.2	88.7	138.4	15.7
EfficientNet-B4	92.1	90.3	19.3	4.5
ViT-B/16	93.8	91.5	86.6	17.2
Swin-Tiny	95.4	93.1	28.3	4.9
Proposed	97.8	93.5	25.7	5.2

**Table 8:** Cross-architecture performance comparison.

Serial No.	Multispectral Six-Channel Images	RGB Three-Channel Image	OB-RF	OB-SVM	Improved ResNet Network	Test Set Accuracy (%)
1	√		√			93.6
2	√			√		92.9
3	√				√	97.8
4		√	√			91.2
5		√		√		92.4
6		√			√	92.3

## Data Availability

The dataset supporting the conclusions of this article is available in the github repository at https://github.com/zyl-mwy/lzp_code/blob/main/README.md (accessed on 24 February 2025). The code supporting the conclusions of this article is available in the github repository at https://github.com/zyl-mwy/lzp_code (accessed on 24 February 2025).

## Abbreviations

The following abbreviations are used in this manuscript:

UAV	Unmanned Aerial Vehicles
LULC	Land Use/Land Cover
CNNs	Convolutional Neural Networks
BN	Batch Normalization
RS1	The Dimension-Altering Residual Structure
RS2	The Dimension-Preserving Residual Structure
P	Precision
R	Recall
OA	Overall Accuracy
OB-RF	Object-Based Random Forest
OB-SVM	Object-Based Support Vector Machine
